# Short-Term Results of Ocriplasmin versus Prompt Vitrectomy for Macular Hole. Which Performs Better?

**DOI:** 10.3390/jcm9123972

**Published:** 2020-12-07

**Authors:** Andrea Cacciamani, Pamela Cosimi, Marta Di Nicola, Guido Ripandelli, Fabio Scarinci

**Affiliations:** 1 IRCCS Fondazione Bietti, 00198 Rome, Italy; pamela.cosimi@fondazionebietti.it (P.C.); guido.ripandelli@fondazionebietti.it (G.R.); fabioscarinci@gmail.com (F.S.); 2 Department of Medical, Oral and Biotechnological Sciences, “G. d’Annunzio” University, 66100 Chieti, Italy; marta.dinicola@unich.it

**Keywords:** optical coherence tomography, microperimetry, macular hole, ocriplasmin, pars plana vitrectomy, retinal sensitivity, visual acuity

## Abstract

In this retrospective study, we compared the anatomical and functional changes in patients with vitreomacular traction associated with macular holes between the following groups: (1) Patients who were treated with a single intravitreal injection of ocriplasmin (the OCRIALONE group); (2) those who failed the ocriplasmin treatment and underwent vitrectomy one month later (the OCRIVIT group); and (3) patients who directly underwent par plana vitrectomy (VITREALONE group). A total of 38 patients, 19 in the OCRIALONE group + OCRIVIT group (seven and 12 patients, respectively) and 19 in the VITREALONE group with focal vitreomacular adhesion associated with macular holes were evaluated with spectral domain optical coherence tomography. Functional examinations included best-corrected visual acuity (BCVA) and microperimetry analysis. Visual function changes were compared between the OCRIALONE group + OCRIVIT group and VITREALONE group up to three months. Furthermore, a subgroup analysis compared the OCRIVIT group and the VITREALONE group. BCVA values and the mean retinal sensitivity showed statistically significant improvement in all groups (*p* < 0.001). Specifically, the retinal sensitivity values at the end of the follow-up were significantly higher in the OCRIALONE group + OCRIVIT group than in the VITREALONE group. These functional findings were also confirmed when the statistical analysis was conducted between the OCRIVIT group and the VITREALONE group. Although the OCRIALONE group + OCRIVIT group exhibited faster retinal thinning than the VITREALONE group (*p* = 0.006), the analysis of the OCRIVIT group versus the VITREALONE group did not show any statistically significant difference. The better functional results and similar anatomical findings suggest that ocriplasmin can be used as a first-line treatment, and that prompt pars plana vitrectomy as primary surgery does not provide better outcomes in comparison with pars plana vitrectomy after ocriplasmin injection.

## 1. Introduction

Full-thickness macular hole is a vitreoretinal disease that seriously compromises the connections of the foveal photoreceptors, causing a severe decrease in central visual function [1]. 

Rapid restoration of anatomical integrity is of fundamental importance to prevent progressive visual reduction.

Pars plana vitrectomy (PPV) with the removal of the internal limiting membrane (ILM), in which gas is introduced into the vitreous chamber, still offers the best results for achieving macular hole closure, with percentages that exceed 90% in the post-operative period [2,3]. However, surgical techniques are not without consequences, and present some risks to the retina and the lens [3,4]. PPV with gas introduction is commonly related to a high risk of cataract onset in the follow-up [4,5,6]. In addition, vitrectomy involves the risk, albeit low, of retinal breaks and retinal detachments caused by the surgical maneuvers necessary to induce the posterior detachment of the vitreous [6].

Intravitreal injection of ocriplasmin represents an alternative approach to treat a vitreomacular traction associated with a full-thickness macular hole of less than 400 μm by inducing an enzymatic vitreolysis, with success rates of up to 40% [7,8].

However, the use of ocriplasmin [9] has been criticized because it has a lower success rate in resolving full-thickness macular holes in comparison with standard vitrectomy. This can lead to higher costs and the need to perform PPV after one month in unsolved cases, with a consequent delay in anatomical restoration and recovery of visual function. By contrast, some authors [10] recommend using ocriplasmin as a first-choice treatment for vitreomacular tractions associated with medium-to-small macular holes (<400 μm) because of its association with lower intra- and post-operative risks in comparison with PPV.

Currently, eyes with macular holes are assessed using spectral domain optical coherence tomography (SD-OCT). This imaging technique is considered as essential to better analyze and measure the anatomical changes of the macula and any vitreoretinal interface disorders. Microperimetry can be used to analyze central retinal sensitivity, and it provides reliable and quantitative information about the macular hole [11].

This study aimed to compare the short-term anatomical and functional changes in three groups of patients diagnosed with vitreomacular traction associated with macular hole (<400 μm): those who were either treated with a single intravitreal injection of ocriplasmin (OCRIALONE group) or patients who failed this treatment and underwent vitrectomy one month later (the OCRIVIT group), and, finally, patients who directly underwent PPV (VITREALONE group).

The following question was raised: if the treatment with ocriplasmin for macular hole fails, does PPV as a secondary surgery yield the same outcomes in comparison with patients who underwent vitrectomy as the first choice?

## 2. Materials and Methods

This retrospective study was performed over a one-year study period from January 2019 to January 2020. We analyzed the data of patients from the Department of Ophthalmology, IRCCS Bietti Foundation (Rome, Italy) who underwent ocriplasmin treatment or PPV.

The study was approved by the local ethics committee (ERMLAB01 N° 77/18/FB) and performed in accordance with the ethical standards stated in the Declaration of Helsinki. The first-line treatment for all patients was based on the availability of the ocriplasmin drug.

The inclusion criteria of the study were patients aged at least 18 years old, as well as the presence of focal vitreomacular adhesion, defined as vitreous adhesion to the macula within a 6 mm central retinal field surrounded by elevation of the posterior vitreous cortex associated with macular hole, as seen on SD-OCT.

The exclusion criteria were patients with cataracts with a nuclear opalescence grade > NO1, based on the lens opacities classification system III grading system (LOCS III); [12] patients treated for cataract surgery in the previous three months; those who had proliferative diabetic retinopathy, neovascular age-related macular degeneration, intravitreal injection, retinal vascular occlusion, aphakia, high myopia (more than −6 diopters), glaucoma, vitreous opacification, lenticular or zonular instability, or a history of retinal detachment in either eye; and those with incomplete charts, who were lost to follow-up, or had low quality OCT images.

Examinations included best-corrected visual acuity (BCVA) measured with the Early Treatment for Diabetic Retinopathy Study chart, complete examination by slit lamp biomicroscopy, SD-OCT scan evaluation (Spectralis, version 1.5.12.0; Heidelberg Engineering, Heidelberg, Germany), and microperimetry analysis (Nidek Technologies, Padova, Italy).

### 2.1. Study Design

Forty-two consecutive patients with vitreomacular traction associated with macular hole (<400 μm) were enrolled in this study. A further four patients were excluded because of incomplete charts and low-quality OCT images. (Two patients in the OCRIALONE group, one in the OCRIVIT group, and one in the VITREALONE group).

(1) Baseline: Patients were divided into three groups. a. The OCRIALONE group comprised those who were treated with a single intravitreal injection of ocriplasmin; b. The OCRIVIT group comprised patients who failed this treatment and underwent vitrectomy one month later; and c. the VITREALONE group comprised patients who directly underwent PPV.

A total of 19 injections of ocriplasmin, seven in the OCRIALONE and 12 in the OCRIVIT group, were performed.

(2) Month 0 to 3: During three months of follow-up, differences in the OCRIALONE group + OCRIVIT group vs. the VITREALONE group and OCRIVIT group vs. the VITREALONE group in the mean 4° and 10° central retinal sensitivity, BCVA values, and OCT foveal thickness were explored. The trend until a three-month follow-up was also evaluated for each group.

### 2.2. Surgical Procedure

All intravitreal injections of commercially available ocriplasmin (125 μg in a 0.10-mL volume) were performed according to national and international guidelines [13]. All patients underwent PPV four weeks later if ocriplasmin treatment failed.

One surgeon (A.C.) performed the operative procedure based on a 25-gauge standard three-port PPV that included removal of ILM peeling. A posterior vitreous detachment was induced, if not already present, by applying high aspiration above the optic nerve and lifting the posterior hyaloid. The ILM was peeled using an intraocular dye composed of soluble lutein, brilliant blue, and trypan blue (Kemin Pharmaceutica Unipessoal, LTDA) in all cases, and creation of a 360° ILM flap around the macula hole (MH) rim.

A second stain with the intraocular dye was performed to verify whether ILM peeling was complete, then fluid-air exchange and 20% hexafluoroethane (C_2_F_6_) injection were performed. During air-fluid exchange, the ILM flap was folded as a single layer to bridge tissue dehiscence. After surgery, patients were requested to adopt a face-down position for 4–5 days.

### 2.3. Image Acquisition

The patients’ mean retinal sensitivity (MRS) was tested using a customized radial grid of 36 stimuli covering the central 10° (centered on the fovea), using the following parameters: the time between stimuli was equal to 1 s; the stimulus size was equivalent to Goldman III; the white background was set at 4 asb; and a bright red cross of 2° was used as the fixation target. A 4-2 double staircase strategy was used, and the first stimulus was presented at a level of 10 dB. MRS was calculated in the entire 10° and in the 4° central area. In each patient, microperimetry was performed twice within the same day to rule out potential learning effects, and the second test was used for the analysis. Moreover, patients underwent a brief training session at the beginning of each test. Tropicamide 1% was used to dilate the pupil in the selected eye.

Two graders (AC and FS), blinded to any clinical information or surgical technique, were asked to measure the MH size. The SD-OCT radial scan centered on the MH was obtained by means of an internal software tool to measure MH diameter and inner segment and outer segment (IS/OS) interruption. These measurements were performed manually.

Measurements of the central foveal thickness (CFT) and mean thickness in the central 1 mm diameter area were obtained with an automatic SD-OCT tool after the surgery.

### 2.4. Statistical Analysis

The Shapiro–Wilk test showed a non-normal distribution of the evaluated parameters. Thus, a non-parametric analysis was conducted. The qualitative variables are summarized as frequency and percentage, and quantitative variables are summarized as the median and interquartile range (IQR). The results were reported separately for each of all groups (OCRIALONE, OCRIVIT, and VITREALONE).

The Kruskal–Wallis rank sum test was applied to compare the quantitative variables between the three groups. The Mann–Whitney U test was used to evaluate statistically significant differences between the macro group (OCRIALONE + OCRIVT) and VITREALONE group. Pearson’s chi-square test was applied for qualitative variables. A linear mixed model for repeated measurements was applied to evaluate the effect of each factor (type of treatment and time) and their interaction on the quantitative parameter evaluated. A mixed model is a powerful method for analyzing data from longitudinal studies, in which there are multiple measurements on each participant [14]. This approach allows explicit modeling of the within-person and between-person variation in the outcome, while considering the correlation between repeated measurements on the same individual. A linear mixed model for repeated measurements was used to regress different time-point values on the fixed-effect factors, assuming an unstructured covariance matrix. In all models, a priori contrasts were used to compare the median of different parameters between the OCRIALONE group + OCRIVIT group and the VITREALONE group, as well as between the OCRIVIT group and the VITREALONE group at different time-points or between previous time-point values in either group.

To assess inter- and intra-observer variability for SD-OCT measurements, the intra-class correlation coefficient (ICC) was calculated. All tests were two-sided, and the level of statistical significance was set at *p* < 0.05. All statistical analyses were performed using the R software environment for statistical computing and graphics version 3.5.2 (R Foundation for Statistical Computing, Vienna, Austria).

## 3. Results

The analysis of the results was conducted to simultaneously explore the following: 1. The impact of surgical therapy duration until the three-month follow-up, that is, the “effect of time”; 2. the impact of the type of treatment (OCRIVIT group vs. VITREALONE group), that is, the “effect of treatment”; and 3. the interaction between surgical therapy duration and treatment, that is, the “interaction effect of time and treatment”.

### 3.1. Patient Characteristics

**Figure 1:**
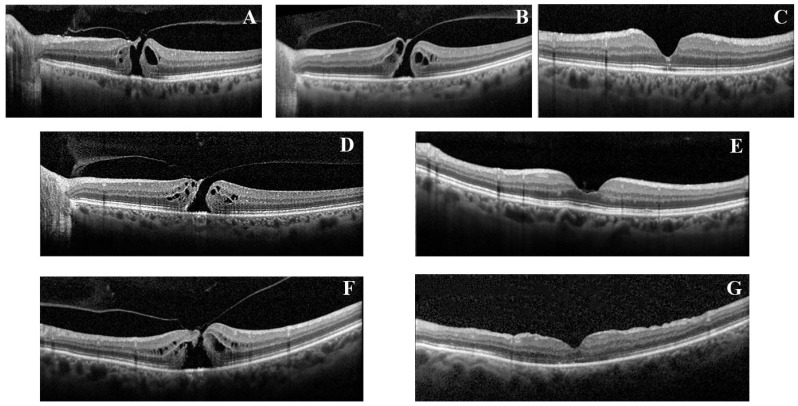
(**A**), baseline optical coherence tomography scan of a patient in the OCRIVIT group who failed the treatment with ocriplasmin (as shown in (**B**) at 1 month follow up) and underwent vitrectomy 1 month later with macular hole closure. Figure (**C**) shows the anatomical result 3 months after surgery. In the middle line, (**D**) shows the baseline characteristics of a patient with macular hole in the OCRIALONE group and (**E**) the macular hole closure after a single ocriplasmin injection 3 months after treatment. Bottom line, (**F**) baseline scan of a patient with macular hole in the VITREALONE and (**G**) shows results 3 months after prompt vitrectomy and gas injection.

Thirty-eight consecutive patients, 19 in the OCRIALONE group + OCRIVIT group (7 and 12 patients, respectively) and 19 in the VITREALONE group were enrolled in this study after meeting the inclusion criteria. All enrolled patients achieved macular hole closure, and no important adverse events occurred. (Figure 1) Baseline clinical characteristics of the groups are described in Table 1, and intergroup anatomical and functional differences were not statistically significant.

In the OCRIALONE group + OCRIVIT group, ocriplasmin injection was the first intervention experienced in all 19 patients. Seven out of these 19 patients (37%) achieved MH closure within 28 days (OCRIALONE group); the remaining 12 patients (63%) underwent subsequent vitrectomy with ILM peeling for MH repair one month later, and achieved MH closure within 30 days after vitrectomy (OCRIVIT group). The eyes of 13 patients (seven treated with ocriplasmin and six vitrectomy-treated patients) were phakic, while the eyes of the remaining six patients in this group were pseudophakic.

In the VITREALONE group, surgery was performed as the first treatment in 19 patients with 100% MH closure within 30 days after vitrectomy. Eight eyes were phakic and 11 eyes were pseudophakic.

All 38 patients did not show a clinically significant cataract (LOCS III) during the entire three-month follow-up, and phacoemulsification surgery was not performed.

### 3.2. Functional and Structural Findings before and after Surgery

BCVA values showed statistically significant improvement in both groups during the entire follow-up period (*p* < 0.001), but there was no statistically significant difference between the groups in terms of treatment (*p* = 0.186) and the interaction effect of time and treatment (*p* = 0.877) (see Table 2).

The mean retinal sensitivity in the 4° and 10° central areas showed statistically significant improvement in all groups at the end of the follow-up period (*p* < 0.001). The retinal sensitivity values at the end of the follow-up were significantly higher in the OCRIALONE group + OCRIVIT group than those in the VITREALONE group (*p* = 0.008 and *p* = 0.017 for 4° and 10°, respectively).

In addition, fixation stability increased significantly in all groups at three months after surgery compared to preoperative values. After three months, the number of patients with stable fixation increased from two to five, four to seven, and three to 13 in the OCRIALONE group, OCRIVIT group, and the VITREALONE group, respectively (*p* < 0.001), with no statistically significant difference between groups in terms of treatment and the interaction effect of time and treatment in the analyses comparing the OCRIALONE + OCRIVIT groups vs. VITREALONE group, or in the analysis comparing the OCRIVIT group vs. VITREALONE group (see Table 2 and Table 3).

Analysis of variance showed a statistically significant reduction in mean foveal thickness in all groups, with a statistically significant difference between groups (*p* = 0.048). Specifically, the OCRIALONE group + OCRIVIT group exhibited faster retinal thinning than the VITREALONE group (*p* = 0.006). However, when considering the OCRIVIT group vs. VITREALONE group, the type of treatment did not result in a statistically significant difference. The details of the analysis are presented in Table 2 and Table 3.

The ICC value for the measurements was 0.905.

## 4. Discussion

In the present study, MH treatment was successful in all 38 patients, regardless of the surgical technique. Nineteen patients were treated with intravitreal injection with ocriplasmin, plus vitrectomy if macular hole closure was not achieved, and 19 patients were treated at presentation with vitrectomy alone.

The clinical anatomical and functional findings in all groups appeared similar at baseline. At the one-month follow-up, BCVA improved significantly only in the OCRIALONE group + OCRIVIT group, while at the end of the follow-up period, the BCVA improvement was similar in all groups regardless of the type of treatment. Moreover, the mean retinal sensitivity at 4° and 10° significantly improved after treatment in all groups. Despite the lack of difference in BCVA measure between groups at the end of the follow-up period, the microperimetry evaluation showed that the retinal sensitivity in the OCRIALONE group + OCRIVIT group was improved as early as the first- and third-month follow-up for the 4° or 10° area, respectively. Conversely, in the VITREALONE group, the retinal sensitivity improved in both the 4° and 10° areas only at the end of the follow-up period. Also, with the exclusion of the OCRIALONE group, in the analysis exploring the OCRIVIT group vs. VITREALONE group, the functional findings were comparable.

As shown in previous studies [15,16], these results indicate that foveal and perifoveal sensitivity analyzed by means of microperimetry, compared to visual acuity, better quantifies functional improvement following treatment, even in patients diagnosed with MH. It is interesting to speculate that these findings in the OCRIALONE group + OCRIVIT group might depend on the fact that less Müller cell trauma, which is usually caused by internal limiting membrane peeling during vitrectomy, was observed in the nine patients who did not have vitrectomy. Furthermore, in the remaining 12 patients in the OCRIVIT group, the ocriplasmin injection, due to its enzymatic activity, might have induced liquefaction and separation of the vitreous cortex from the internal retina and facilitated peeling during vitrectomy. In line with this, a previous study demonstrated that, unlike conventionally vitrectomized eyes, the use of an intravitreal injection of plasmin adjuvant 30 min prior to vitrectomy resulted in an ILM less adherent to the retina, consistent with complete PVD (posterior vitreous detachment) [17]. A study by Asami et al. [18] showed that eyes with diabetic macular edema and injected with autologous plasmin before vitrectomy presented a smooth surface on the vitreous side of the ILM and only sparse vitreous remnants. These findings also highlighted that remnants of vitreous strands are more effectively removed from the inner surface of the ILM with plasmin-assisted vitrectomy. Furthermore, other studies [19,20,21] showed that in patients with MH, the use of plasmin enzyme in the vitreous cavity, the creation of a posterior vitreous separation, and perhaps the activation of endogenous factors around the macular hole can reduce or eliminate the need for manipulation of vitreous collagen, in turn reducing the need for meticulous work close to the retinal surface.

Finally, inducing a posterior vitreous separation and liquefaction may increase exposure of oxygen to the retina [22] and improve the health status of the photoreceptors one month before the surgical trauma in the OCRIVIT group. This may account for the better functional outcomes seen in the OCRIVIT group compared to the VITREALONE group.

Looking at the BCVA values, previous reports [23,24,25] did not find a real risk in delaying the closure of the MH by one month in patients treated with ocriplasmin plus vitrectomy compared to the vitrectomy alone group. Our findings confirm that delaying the vitrectomy in cases that had undergone unsuccessful vitreolytic treatment with ocriplasmin did not affect the clinical success achieved in terms of visual function, and highlighted that these patients might experience better improvement in retinal sensitivity at three months after surgery in comparison to those for which vitrectomy is selected as first-line treatment. However, a longer follow-up is needed to confirm if these preliminary findings indeed culminate in a true advantage in clinical practice.

Fixation stability improved in all groups at the end of the follow-up, with 12 (63.2%, specifically 7 patients belong to the OCRIVIT group) in the OCRIALONE group + OCRIVIT group and 13 patients (68.4%) in the VITREALONE group. Looking at the structural OCT parameters, the OCRIALONE group + OCRIVIT group showed faster retinal thinning as early as the first month of follow-up. However, the analysis of the OCRIVIT group vs. VITREALONE group did not quite reach the level of significance for the type of treatment. Furthermore, the central retinal thickness at the end of the follow-up period was similar between groups. The reason for these anatomical changes and the subsequent retinal remodeling in these patients could not be established in this study. Further studies using adaptive optics tools could better clarify this. However, the retina is an advanced neuronal and vascular complex, and the restoration of the neurovascular connections might allow for better visual recovery in a short-term analysis [26].

Cataract surgery was not performed during the study. Since progressive lens opacification arises after vitrectomy, microperimetry tests beyond three months were not performed in this study. In fact, a cataract significantly influences retinal sensitivity values, and in these patients, microperimetry evaluation might be less accurate [27,28]. Conversely, a longer follow-up period may be of interest to explore the possibility that retinal sensitivity could be similar, even when different treatments are used.

In a previous study, a comparison of the anatomical and visual outcomes of patients with bilateral MH, who were treated with PPV in one eye and intravitreal ocriplasmin in the fellow eye, showed that the treatments resulted in similar levels of visual acuity improvement [25]. However, on post-operative SD-OCT examination, ellipsoid zone disruption was more frequent in the vitrectomized eyes [25]. Moreover, in a limited case series, Benarous et al. [23] showed that failure of treatment with ocriplasmin does not compromise the subsequent anatomical and functional success of postponed vitrectomy for macular holes.

The main limitations of this study were its retrospective design, the lack of randomization, and the relatively small sample size. In addition, in most patients, the MHs were smaller than 350 µm, and the follow-up period was limited to three months. A longer follow-up is also important to confirm the higher improvement of retinal sensitivity found in the OCRIVIT group.

From a clinical point of view, the better and faster functional and anatomical results in the OCRIVIT group suggest that ocriplasmin can be used as first-line treatment. In fact, ocriplasmin results in less trauma and fewer consequences on the retinal structures with respect to vitrectomy. Moreover, since it is a less invasive treatment, ocriplasmin may offer a suitable and safe alternative for patients with a contraindication to gas injection, including air travel, difficulties in postoperative positioning, and claustrophobic patients who would not undergo general anesthesia [29]. However, if ocriplasmin is not effective, we should still consider the need to expose these patients to a second procedure.

## 5. Conclusions

Ocriplasmin can avoid vitrectomy in at least a quarter of well-selected patients, and prompt PPV as primary surgery does not provide better outcomes in comparison with PPV after ocriplasmin injection. Visual acuity evaluation alone appears to underestimate the functional outcome obtained either with ocriplasmin alone or in combination with PPV. However, only a randomized controlled study with a larger sample and a longer follow-up period can provide a definitive answer and confirm these findings.

## Figures and Tables

**Table 1:** Baseline characteristics of patients enrolled.

Variable	OCRIALONE (*n* = 7)	OCRIVIT (*n* = 12)	VITREALONE (*n* = 19)	*p*-Value ^a^	OCRIALONE + OCRIVT (*n* = 19)	*p*-Value ^b^
GENDER, n (%)				0.906 ^1^		0.744 ^1^
Female	4 (57.1%)	6 (50.0%)	11 (57.9%)		10 (52.6%)	
Male	3 (42.9%)	6 (50.0%)	8 (42.1%)		9 (47.4%)	
Age (yr), Median (IQR)	62.0 (58.0, 69.0)	67.0 (64.2, 70.2)	65.0 (62.0, 71.5)	0.652	67.0 (58.5, 70.0)	0.792
BCVA (LogMAR), Median (IQR)	0.4 (0.3, 0.6)	0.6 (0.5, 0.8)	0.6 (0.4, 0.8)	0.188	0.5 (0.3, 0.7)	0.595
ETDRS, Median (IQR)	65.0 (57.5, 70.0)	52.5 (43.8, 59.8)	55.0 (47.5, 65.0)	0.188	58.0 (50.0, 67.5)	0.595
IS/OS size (µ), Median (IQR)	632.0 (594.0, 702.0)	647.0 (564.7, 771.7)	657.0 (508.0, 870.0)	0.902	632.0 (594.0, 762.0)	
MH size (µ), Median (IQR)	300.0 (272.5, 360.0)	299.0 (277.8, 363.8)	340.0 (270.0, 374.0)	0.956	300.0 (277.5, 364.5)	0.770
Retinal Sensitivity 4° (dB), Median (IQR)	13.0 (12.1, 14.1)	11.8 (8.2, 15.5)	11.0 (8.5, 13.0)	0.312	12.2 (10.0, 14.6)	0.193
Retinal Sensitivity 10° (dB), Median (IQR)	14.9 (10.4, 16.0)	15.9 (11.4, 19.1)	13.4 (11.4, 16.1)	0.537	15.5 (11.3, 17.7)	0.540
Fixation Stability, n (%)				0.828 ^1^		0.416 ^1^
S	2 (28.6%)	4 (33.3%)	3 (15.7%)		6 (31.6%)	
RU	3 (42.8%)	4 (33.3%)	9 (47.5%)		7 (36.8%)	
U	2 (28.6%)	4 (33.3%)	7 (36.8%)		6 (31.6%)	

yr, years; BCVA, best corrected visual acuity; ETDRS, Early Treatment for Diabetic Retinopathy Study; IS/OS, inner segment/ outer segment; MH, macular hole; dB, decibel; S, stable; RU, relatively unstable; U, unstable. ^a^ *p*-value derived to Kruskal-Wallis rank sum test between three groups; ^b^ *p*-value derived to Mann-Whitney test vs. VITREALONE group; ^1^ Pearson’s Chi-squared test.

**Table 2:** Functional and structural findings before and after surgery in the OCRIALONE group + OCRIVIT group vs. VITREALONE.

Variable	OCRIALONE + OCRIVIT Group (*n* = 19, 7 + 12, Respectively)			VITREALONE Group (*n* = 19)			*p*-Value		
	Baseline	1 Month	3 Months	Baseline	1 Month	3 Months	Time ^a^	Group ^b^	Interaction ^c^
BCVA(LogMAR)	0.5 (0.3, 0.7)	0.2 (0.1, 0.4) *	0.2 (0.1, 0.3)	0.6 (0.4, 0.8)	0.4 (0.3, 0.8)	0.2 (0.1, 0.4) *	**<0.001**	0.186	0.877
ETDRS	58.0 (50.0, 67.5)	73.0 (65.0, 79.5) *	75.0 (67.0, 78.0)	55.0 (47.5, 65.0)	65.0 (42.5, 70.0)	72.0 (65.0, 76.0) *	**<0.001**	0.184	0.840
Retinal Sensitivit 4° (dB)	12.2 (10.0, 14.6)	17.0 (15.5, 22.5) *	19.0 (16.0, 23.5) *	11.0 (8.5, 13.0)	12.0 (11.0, 15.5) *	18.0 (12.0, 20.0) *	**<0.001**	**0.008**	0.235
Retinal Sensitivity 10° (dB)	15.5 (11.3, 17.7)	19.5 (17.0, 23.9) *	21.3 (18.8, 24.1) *	13.4 (11.4, 16.1)	14.7 (11.7, 16.9)	19.5 (14.3, 23.0) *	**<0.001**	**0.017**	0.459
Foveal Thickness (µ)	-	228.5 (188.2, 289.8)	231.0 (181.5, 284.0)	-	274.0 (207.0, 331.8)	229.0 (199.5, 266.5) *	**0.004**	**0.048**	**0.006**
Fixation Stability, n (%)							**0.001**	0.297	0.367
S	6 (31.6%)	12 (63.2%)	12 (63.2%)	3 (15.8%)	8 (42.1%)	13 (68.4%)			
RU	7 (36.8%)	4 (21.1%)	6 (31.6%)	9 (47.4%)	5 (26.3%)	3 (15.8%)			
U	6 (31.6%)	3 (15.8%)	1 (5.3%)	7 (36.8%)	6 (31.6%)	3 (15.8%)			

Bolded *p*-values are significant after FDR correction. Data are expressed as median and interquartile range (IQR). BCVA, best corrected visual acuity; ETDRS, Early Treatment for Diabetic Retinopathy Study; dB, decibel; S, stable; RU, relatively unstable; U, unstable. ^a^ The effect of time for each variable; the differences were tested between the means of the two groups at different follow-up controls. ^b^ The effect of group for each variable; the differences have been tested between the means of the OCRIALONE group + OCRIVIT group at three time points (baseline, one and three months post-surgery) and the means of the VITREALONE group at three follow-up controls. ^c^ Probability that the effects of time are greater in one distinct group (interaction time*group). * *p* < 0.05 contrast analysis *p*-value refers to the previous time point.

**Table 3:** Functional and structural findings before and after surgery in the OCRIVIT group vs. VITREALONE group.

Variable	OCRIVIT Group (*n* = 12)			VITREALONE Group (*n* = 19)			*p*-Value		
	Baseline	1 Month	3 Months	Baseline	1 Month	3 Months	Time ^a^	Group ^b^	Interaction ^c^
BCVA(LogMAR)	0.7 (0.4–0.9)	0.3 (0.1–0.6) *	0.3 (0.1–0.7)	0.6 (0.4, 0.8)	0.4 (0.3, 0.8)	0.2 (0.1, 0.4) *	**<0.001**	0.675	0.083
ETDRS	52.5 (41.2–63.2)	69.0 (55.0–80.2) *	72.5 (52.5–78.0)	55.0 (47.5, 65.0)	65.0 (42.5, 70.0)	72.0 (65.0, 76.0) *	**<0.001**	0.659	0.088
Retinal Sensitivity 4° (dB)	11.8 (6.7–16.5)	16.0 (14.2–24.2) *	18.0 (14.5–25.0) *	11.0 (8.5, 13.0)	12.0 (11.0, 15.5) *	18.0 (12.0, 20.0) *	**<0.001**	**0.047**	0.498
Retinal Sensitivity 10° (dB)	15.9 (11.3–19.9)	20.0 (15.0–25.8) *	21.1 (17.4–25.3)	13.4 (11.4, 16.1)	14.7 (11.7, 16.9)	19.5 (14.3, 23.0) *	**<0.001**	**0.033**	0.956
Foveal Thickness (µ)	-	213.0 (190.2–301.7)	237.5 (178.2–304.0)	-	274.0 (207.0, 331.8)	229.0 (199.5, 266.5) *	**0.030**	0.105	**0.008**
Fixation Stability, n (%)							**0.001**	0.571	0.571
S	4 (33.3%)	7 (58.3%)	7 (58.3%)	3 (15.8%)	8 (42.1%)	13 (68.4%)			
RU	4 (33.3%)	2 (16.7%)	4 (33.3%)	9 (47.4%)	5 (26.3%)	3 (15.8%)			
U	4 (33.3%)	3 (25.0%)	1 (8.3%)	7 (36.8%)	6 (31.6%)	3 (15.8%)			

BCVA, best corrected visual acuity; ETDRS, Early Treatment for Diabetic Retinopathy Study; dB, decibel; S, stable; RU, relatively unstable; U, unstable. Bolded *p*-values are significant after FDR correction; Effect of time for each variable; the differences have been tested between the means of the two groups at different follow-up control. ^a^ The effect of time for each variable; the differences were tested between the means of the two groups at different follow-up controls. ^b^ The effect of group for each variable; the differences have been tested between the means of the OCRIALONE group + OCRIVIT group at three time points (baseline, one and three months post-surgery) and the means of the VITREALONE group at three follow-up controls. ^c^ Probability that the effects of time are greater in one distinct group (interaction time*group). * *p* < 0.05 contrast analysis *p*-value refers to the previous time point.
